# Ethyl 8-amino-6-bromoimidazo[1,2-*a*]pyridine-2-carb­oxy­late

**DOI:** 10.1107/S1600536811017077

**Published:** 2011-05-11

**Authors:** Siham Dahmani, Youssef Kandri Rodi, Santiago V. Luis, El Mokhtar Essassi, Lahcen El Ammari

**Affiliations:** a Laboratoire de Chimie Organique Appliquée, Université Sidi Mohamed Ben Abdallah, Faculté des Sciences et Techniques, Route d’Immouzzer, BP 2202 Fès, Morocco; b Departamento de Quimica Inorganica & Organica, ESTCE, Universitat Jaume I, E-12080 Castellon, Spain; c Laboratoire de Chimie Organique Hétérocyclique URAC21, Faculté des Sciences, Université Mohammed V-Agdal, Avenue Ibn Battouta, BP 1014, Rabat, Morocco; d Laboratoire de Chimie du Solide Appliquée, Faculté des Sciences, Université Mohammed V-Agdal, Avenue Ibn Battouta, BP 1014, Rabat, Morocco

## Abstract

There are two independent mol­ecules in the asymmetric unit of the title compound, C_10_H_10_BrN_3_O_2_, which are linked by N—H⋯O and C—H⋯O hydrogen bonds. The fused ring systems in both mol­ecules are nearly planar with maximum deviations of 0.001 (3) and 0.029 (3) Å. All non-H atoms of the first mol­ecule are approximately co-planar whereas in the second mol­ecule, the ethyl group is almost perpendicular to the imidazo[1,2-*a*]pyridine system, the C—O—C—C torsion angles in the carb­oxy­lic acid ethyl group  being −179.8 (4) and 112.1 (5)°, respectively.

## Related literature

For the biological activity of imidazo[1,2-*a*]pyridine derivatives, see: Anderson *et al.* (2003 ▶); Trapani *et al.* (2003 ▶); Gueiffier *et al.* (1998 ▶); Mavel *et al.* (2002 ▶). For their pharmacological activity, see: Rival *et al.* (1992 ▶); Rupert *et al.* (2003 ▶); Katritzky *et al.* (2003 ▶).
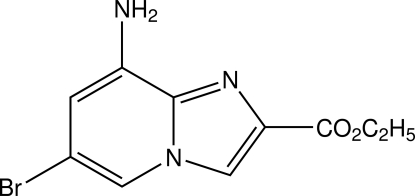


## Experimental

### 

#### Crystal data

C_10_H_10_BrN_3_O_2_
*M* *_r_* = 284.12
Monoclinic, 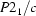

*a* = 8.366 (2) Å
*b* = 11.842 (3) Å
*c* = 22.743 (5) Å
β = 98.328 (6)°
*V* = 2229.3 (8) Å^3^
*Z* = 8
Mo *K*α radiation
μ = 3.68 mm^−1^
*T* = 292 K
0.44 × 0.19 × 0.17 mm

#### Data collection

Bruker APEXII CCD diffractometer
Absorption correction: multi-scan (*SADABS*; Bruker, 2009 ▶) *T*_min_ = 0.437, *T*_max_ = 0.535
13222 measured reflections
4555 independent reflections
3156 reflections with *I* > 2σ(*I*)
*R*_int_ = 0.032

#### Refinement

*R*[*F* ^2^ > 2σ(*F* ^2^)] = 0.041
*wR*(*F* ^2^) = 0.116
*S* = 1.03
4555 reflections
291 parameters
H-atom parameters constrained
Δρ_max_ = 0.70 e Å^−3^
Δρ_min_ = −0.51 e Å^−3^

### 

Data collection: *APEX2* (Bruker, 2009 ▶); cell refinement: *SAINT-Plus* (Bruker, 2009 ▶); data reduction: *SAINT-Plus*; program(s) used to solve structure: *SHELXS97* (Sheldrick, 2008 ▶); program(s) used to refine structure: *SHELXL97* (Sheldrick, 2008 ▶); molecular graphics: *ORTEP-3 for Windows* (Farrugia, 1997 ▶); software used to prepare material for publication: *SHELXL97*.

## Supplementary Material

Crystal structure: contains datablocks I, global. DOI: 10.1107/S1600536811017077/dn2683sup1.cif

Structure factors: contains datablocks I. DOI: 10.1107/S1600536811017077/dn2683Isup2.hkl

Supplementary material file. DOI: 10.1107/S1600536811017077/dn2683Isup3.cml

Additional supplementary materials:  crystallographic information; 3D view; checkCIF report

## Figures and Tables

**Table 1:** Hydrogen-bond geometry (Å, °)

*D*—H⋯*A*	*D*—H	H⋯*A*	*D*⋯*A*	*D*—H⋯*A*
N3—H3*A*⋯N5^i^	0.86	2.47	3.300 (4)	161
N3—H3*B*⋯O3^ii^	0.86	2.38	3.055 (5)	135
N6—H6*B*⋯O2	0.85	2.34	3.096 (4)	147
N6—H6*A*⋯O1^iii^	0.86	2.58	3.183 (4)	129
N6—H6*A*⋯N2^iii^	0.86	2.60	3.388 (4)	154
C5—H5⋯O4	0.93	2.26	3.074 (4)	146

Symmetry codes: (i) 
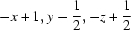
; (ii) 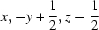; (iii) 
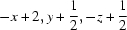
.

**Fig. 1.**
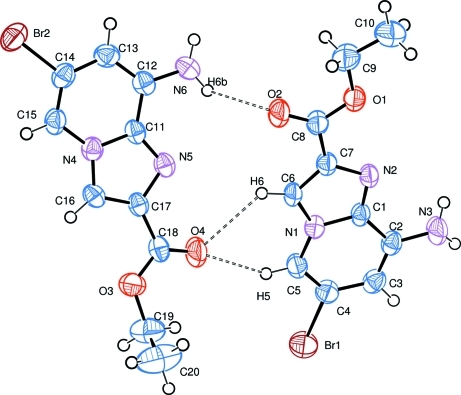
: Plot of of the two molecules building the asymmetric unit of the title compound with the atom-labelling scheme. Displacement ellipsoids are drawn at the 50% probability level. H atoms are represented as small circles of arbitrary radii. Hydrogen bonds are shown as dashed lines.

**Fig. 2.**
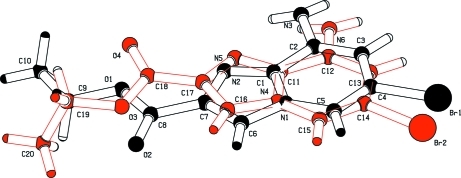
View showing the fitting of the two molecules building the asymmetric unit of the title compound.

## supplementary crystallographic information

### Comment

Imidazo[1,2-*a*]pyridine derivatives are important intermediates in organic synthesis, especially in the synthesis of biologically active and medicinally useful agents. For instance, they are widely used in the synthesis of cyclin- dependent kinases (CDK) inhibitors (Anderson *et al.*, 2003), anticonvulsant agents, (Trapani *et al.*, 2003) and antiviral agents (Gueiffier *et al.*,1998; Mavel *et al.*,2002).

Derivatives containing the imidazo[1,2-*a*]- pyridine ring system have been shown to possess a broad range of useful pharmacological activities, including antibacterial, (Rival *et al.*, 1992) and anti-inflammatory properties (Rupert *et al.*, 2003). Drug formulations containing imidazo[1,2-*a*]pyridines currently available on the market include alpidem (anxiolytic), zolpidem (hypnotic), and zolimidine (antiulcer) (Katritzky *et al.*,2003).

In this work, we report a novel and efficient method for the synthesis of 6-bromo-8-amino-imidazo[1,2-*a*]pyridine *via* the treatment of ethyl bromopyruvate with 5-bromo-2,3-diaminopyridine in the presence of NaHCO_3_ in ethanol at reflux in 65% yield (scheme1).

The Plot of the two molecules building the asymmetric unit of the 8-Amino-6- bromo-imidazo[1,2-*a*]pyridin-2-carboxylic acid ethyl ester is shown in Fig.1. The two fused five and six-membered rings belonging to each molecule are nearly planar with the maximum deviation of -0.001 (3)Å from C7 and 0.029 (3)Å from C17. The dihedral angle between them is 26.06 (11)°. The ethyl group is almost perpendicular to the imidazo[1,2-*a*]pyridine system, in the second molecule, as indicated by the torsion angle C18–O3–C19–C20 of 112.1 (5)° (see Fig.2). The first molecule is approximately planar and the torsion angle C—O—C—C is in the range of -179.8 (4)°. In the crystal, the molecules are linked by intermolecular N—H···O and C—H···O hydrogen bonds building a three dimensionnal network (Table 1).

### Experimental

A mixture of ethyl bromopyruvate (0.3 ml; 2.35 mmol), 5-bromo-2,3-diaminopyridine (0.5 g, 2.35 mmol) and NaHCO_3_ (0.22 g, 2.35 mmol) in ethanol was stirred at reflux for the appropriate time. After completion of the reaction, as indicated by TLC, The solution was extracted with dichloromethane and the organic layer was dried over anhydrous Na_2_SO_4_. Evaporation of the solvent followed by recrystallization in hexane afforded yellow crystals of the title compound.

### Refinement

H atoms were located in a difference map and treated as riding with C—H = 0.93 Å, 0.97, Å, 0.96 Å, and 0.86 Å for aromatic, methylene, methyl and –NH respectively. All H atoms with *U*_iso_(H) = 1.2 *U*_eq_ (aromatic, methylene, –NH) and *U*_iso_(H) = 1.5 *U*_eq_(methyl). H atoms attached to amino groups were located in difference Fourier map and their coordinates were initially refined using N-H restraints (0.86Å with s.u. of 0.01) with *U*_iso_(H) = 1.2 *U*_eq_ (N). In the last cycles of refinement, they were treated as riding on their parent N atoms.

### Crystal data

**Table:**

C_10_H_10_BrN_3_O_2_	*F*(000) = 1136
*M**_r_* = 284.12	*D*_x_ = 1.693 Mg m^−^^3^
Monoclinic, *P*2_1_/*c*	Melting point: 414(2) K
Hall symbol: -P 2ybc	Mo *K*α radiation, λ = 0.71073 Å
*a* = 8.366 (2) Å	Cell parameters from 4555 reflections
*b* = 11.842 (3) Å	θ = 1.8–26.8°
*c* = 22.743 (5) Å	µ = 3.68 mm^−^^1^
β = 98.328 (6)°	*T* = 292 K
*V* = 2229.3 (8) Å^3^	Fiber, yellow
*Z* = 8	0.44 × 0.19 × 0.17 mm

### Data collection

**Table:**

Bruker APEXII CCD diffractometer	4555 independent reflections
Radiation source: fine-focus sealed tube	3156 reflections with *I* > 2σ(*I*)
graphite	*R*_int_ = 0.032
φ and ω scans	θ_max_ = 26.8°, θ_min_ = 1.8°
Absorption correction: multi-scan (*SADABS*; Bruker, 2009)	*h* = −10→10
*T*_min_ = 0.437, *T*_max_ = 0.535	*k* = −11→14
13222 measured reflections	*l* = −24→28

### Refinement

**Table:**

Refinement on *F*^2^	Primary atom site location: structure-invariant direct methods
Least-squares matrix: full	Secondary atom site location: difference Fourier map
*R*[*F*^2^ > 2σ(*F*^2^)] = 0.041	Hydrogen site location: inferred from neighbouring sites
*wR*(*F*^2^) = 0.116	H-atom parameters constrained
*S* = 1.03	*w* = 1/[σ^2^(*F*_o_^2^) + (0.0571*P*)^2^ + 1.0629*P*] where *P* = (*F*_o_^2^ + 2*F*_c_^2^)/3
4555 reflections	(Δ/σ)_max_ = 0.001
291 parameters	Δρ_max_ = 0.70 e Å^−^^3^
0 restraints	Δρ_min_ = −0.51 e Å^−^^3^

### Special details

**Table:**

Geometry. All esds (except the esd in the dihedral angle between two l.s. planes) are estimated using the full covariance matrix. The cell esds are taken into account individually in the estimation of esds in distances, angles and torsion angles; correlations between esds in cell parameters are only used when they are defined by crystal symmetry. An approximate (isotropic) treatment of cell esds is used for estimating esds involving l.s. planes.
Refinement. Refinement of *F*^2^ against ALL reflections. The weighted *R*-factor *wR* and goodness of fit *S* are based on *F*^2^, conventional *R*-factors *R* are based on *F*, with *F* set to zero for negative *F*^2^. The threshold expression of *F*^2^ > 2σ(*F*^2^) is used only for calculating *R*-factors(gt) *etc*. and is not relevant to the choice of reflections for refinement. *R*-factors based on *F*^2^ are statistically about twice as large as those based on *F*, and *R*- factors based on ALL data will be even larger.

### Fractional atomic coordinates and isotropic or equivalent isotropic displacement parameters (Å^2^)

**Table:**

	*x*	*y*	*z*	*U*_iso_*/*U*_eq_	
C1	0.6311 (4)	0.1790 (2)	0.24000 (15)	0.0364 (7)	
C2	0.5137 (4)	0.1042 (3)	0.20858 (16)	0.0424 (8)	
C3	0.3848 (4)	0.0737 (3)	0.23542 (17)	0.0463 (8)	
H3	0.3073	0.0248	0.2162	0.056*	
C4	0.3685 (4)	0.1156 (3)	0.29203 (17)	0.0457 (8)	
C5	0.4772 (4)	0.1854 (3)	0.32284 (16)	0.0458 (8)	
H5	0.4651	0.2114	0.3605	0.055*	
C6	0.7346 (4)	0.2859 (3)	0.31519 (15)	0.0407 (7)	
H6	0.7531	0.3244	0.3512	0.049*	
C7	0.8290 (4)	0.2868 (3)	0.27069 (15)	0.0399 (7)	
C8	0.9804 (4)	0.3528 (3)	0.27430 (17)	0.0442 (8)	
C9	1.2055 (5)	0.4046 (4)	0.2303 (2)	0.0646 (11)	
H9A	1.2805	0.3841	0.2653	0.078*	
H9B	1.1815	0.4845	0.2327	0.078*	
C10	1.2790 (6)	0.3820 (4)	0.1765 (2)	0.0738 (13)	
H10A	1.3755	0.4263	0.1775	0.111*	
H10B	1.2041	0.4018	0.1420	0.111*	
H10C	1.3056	0.3033	0.1750	0.111*	
N1	0.6074 (3)	0.2165 (2)	0.29548 (13)	0.0391 (6)	
N2	0.7654 (3)	0.2206 (2)	0.22339 (12)	0.0387 (6)	
N3	0.5421 (4)	0.0657 (3)	0.15447 (15)	0.0609 (9)	
H3A	0.4634	0.0338	0.1320	0.073*	
H3B	0.6048	0.1043	0.1351	0.073*	
O1	1.0571 (3)	0.33902 (19)	0.22826 (11)	0.0493 (6)	
O2	1.0266 (3)	0.4127 (2)	0.31636 (13)	0.0619 (7)	
Br1	0.18490 (5)	0.07406 (4)	0.32785 (2)	0.06942 (17)	
C11	0.8701 (4)	0.5979 (3)	0.45987 (15)	0.0382 (7)	
C12	0.9608 (4)	0.6732 (3)	0.42770 (16)	0.0422 (8)	
C13	1.0265 (4)	0.7670 (3)	0.45760 (17)	0.0472 (8)	
H13	1.0798	0.8209	0.4379	0.057*	
C14	1.0129 (4)	0.7813 (3)	0.51807 (17)	0.0450 (8)	
C15	0.9362 (4)	0.7088 (3)	0.54986 (17)	0.0462 (8)	
H15	0.9324	0.7195	0.5901	0.055*	
C16	0.7710 (4)	0.5322 (3)	0.53803 (16)	0.0418 (8)	
H16	0.7459	0.5214	0.5762	0.050*	
C17	0.7243 (3)	0.4671 (3)	0.48894 (15)	0.0367 (7)	
C18	0.6206 (4)	0.3662 (3)	0.48367 (16)	0.0432 (8)	
C19	0.4773 (5)	0.2335 (4)	0.5359 (2)	0.0772 (14)	
H19A	0.3926	0.2537	0.5590	0.093*	
H19B	0.4272	0.2181	0.4956	0.093*	
C20	0.5572 (7)	0.1346 (5)	0.5605 (4)	0.118 (2)	
H20A	0.4790	0.0766	0.5638	0.177*	
H20B	0.6147	0.1514	0.5991	0.177*	
H20C	0.6320	0.1091	0.5351	0.177*	
N4	0.8630 (3)	0.6170 (2)	0.51890 (13)	0.0399 (6)	
N5	0.7852 (3)	0.5068 (2)	0.43990 (12)	0.0400 (6)	
N6	0.9732 (4)	0.6470 (3)	0.37068 (14)	0.0565 (8)	
H6A	1.0318	0.6877	0.3512	0.068*	
H6B	0.9494	0.5790	0.3605	0.068*	
O3	0.5891 (3)	0.3286 (2)	0.53566 (12)	0.0551 (6)	
O4	0.5670 (3)	0.3250 (3)	0.43682 (13)	0.0718 (8)	
Br2	1.11787 (5)	0.90661 (3)	0.55921 (2)	0.06673 (16)	

### Atomic displacement parameters (Å^2^)

**Table:**

	*U*^11^	*U*^22^	*U*^33^	*U*^12^	*U*^13^	*U*^23^
C1	0.0447 (17)	0.0342 (16)	0.0290 (19)	0.0055 (13)	0.0002 (13)	−0.0018 (13)
C2	0.0483 (19)	0.0406 (18)	0.036 (2)	0.0027 (14)	−0.0025 (15)	−0.0035 (14)
C3	0.0489 (19)	0.0436 (19)	0.043 (2)	−0.0036 (14)	−0.0038 (15)	−0.0022 (15)
C4	0.0421 (18)	0.0477 (19)	0.048 (2)	−0.0002 (14)	0.0078 (15)	−0.0012 (16)
C5	0.0481 (19)	0.052 (2)	0.039 (2)	−0.0039 (15)	0.0119 (15)	−0.0082 (16)
C6	0.0470 (18)	0.0433 (18)	0.032 (2)	−0.0051 (14)	0.0057 (14)	−0.0103 (14)
C7	0.0445 (18)	0.0396 (17)	0.035 (2)	0.0010 (13)	0.0038 (14)	−0.0032 (14)
C8	0.050 (2)	0.0431 (19)	0.039 (2)	0.0005 (15)	0.0071 (15)	−0.0031 (15)
C9	0.050 (2)	0.071 (3)	0.075 (3)	−0.0188 (18)	0.015 (2)	−0.014 (2)
C10	0.073 (3)	0.076 (3)	0.076 (4)	−0.017 (2)	0.022 (2)	0.002 (2)
N1	0.0401 (14)	0.0400 (14)	0.0370 (18)	0.0004 (11)	0.0050 (11)	−0.0060 (12)
N2	0.0459 (15)	0.0400 (14)	0.0299 (16)	0.0022 (11)	0.0046 (11)	−0.0048 (11)
N3	0.071 (2)	0.074 (2)	0.036 (2)	−0.0150 (16)	0.0027 (15)	−0.0158 (15)
O1	0.0495 (13)	0.0516 (14)	0.0492 (17)	−0.0087 (10)	0.0148 (11)	−0.0136 (11)
O2	0.0700 (17)	0.0704 (18)	0.0464 (18)	−0.0226 (13)	0.0126 (13)	−0.0225 (14)
Br1	0.0567 (3)	0.0777 (3)	0.0779 (4)	−0.01732 (19)	0.0233 (2)	−0.0092 (2)
C11	0.0426 (17)	0.0423 (17)	0.0292 (19)	0.0124 (14)	0.0030 (13)	0.0034 (14)
C12	0.0444 (18)	0.0447 (19)	0.037 (2)	0.0086 (14)	0.0035 (14)	0.0056 (15)
C13	0.052 (2)	0.0419 (19)	0.048 (2)	0.0031 (15)	0.0075 (16)	0.0079 (16)
C14	0.0484 (19)	0.0375 (17)	0.047 (2)	0.0041 (14)	−0.0003 (15)	−0.0022 (15)
C15	0.056 (2)	0.0454 (19)	0.035 (2)	0.0031 (15)	0.0003 (15)	−0.0051 (15)
C16	0.0446 (18)	0.0478 (19)	0.033 (2)	0.0039 (14)	0.0062 (14)	0.0050 (15)
C17	0.0349 (15)	0.0437 (17)	0.032 (2)	0.0073 (13)	0.0047 (13)	−0.0007 (14)
C18	0.0396 (17)	0.0526 (19)	0.038 (2)	0.0056 (14)	0.0075 (15)	−0.0054 (16)
C19	0.067 (3)	0.074 (3)	0.089 (4)	−0.018 (2)	0.006 (2)	0.017 (3)
C20	0.096 (4)	0.076 (4)	0.183 (8)	−0.014 (3)	0.026 (4)	0.027 (4)
N4	0.0433 (15)	0.0402 (15)	0.0352 (18)	0.0041 (11)	0.0024 (12)	−0.0005 (11)
N5	0.0412 (14)	0.0463 (15)	0.0318 (17)	0.0057 (12)	0.0029 (11)	0.0002 (12)
N6	0.081 (2)	0.0522 (18)	0.039 (2)	−0.0051 (15)	0.0172 (15)	0.0063 (14)
O3	0.0587 (15)	0.0573 (15)	0.0489 (18)	−0.0115 (12)	0.0064 (12)	0.0049 (12)
O4	0.0805 (19)	0.088 (2)	0.049 (2)	−0.0289 (16)	0.0165 (14)	−0.0289 (15)
Br2	0.0787 (3)	0.0502 (2)	0.0698 (3)	−0.01168 (19)	0.0056 (2)	−0.01129 (19)

### Geometric parameters (Å, °)

**Table:**

C1—N2	1.331 (4)	C11—N5	1.335 (4)
C1—N1	1.379 (4)	C11—N4	1.371 (4)
C1—C2	1.434 (4)	C11—C12	1.437 (5)
C2—C3	1.363 (5)	C12—N6	1.352 (4)
C2—N3	1.365 (5)	C12—C13	1.376 (5)
C3—C4	1.405 (5)	C13—C14	1.407 (5)
C3—H3	0.9300	C13—H13	0.9300
C4—C5	1.348 (5)	C14—C15	1.344 (5)
C4—Br1	1.904 (3)	C14—Br2	1.900 (3)
C5—N1	1.380 (4)	C15—N4	1.389 (4)
C5—H5	0.9300	C15—H15	0.9300
C6—N1	1.368 (4)	C16—C17	1.367 (5)
C6—C7	1.371 (5)	C16—N4	1.374 (4)
C6—H6	0.9300	C16—H16	0.9300
C7—N2	1.374 (4)	C17—N5	1.375 (4)
C7—C8	1.480 (5)	C17—C18	1.472 (5)
C8—O2	1.209 (4)	C18—O4	1.199 (4)
C8—O1	1.315 (4)	C18—O3	1.325 (4)
C9—O1	1.460 (4)	C19—C20	1.422 (7)
C9—C10	1.471 (6)	C19—O3	1.464 (5)
C9—H9A	0.9700	C19—H19A	0.9700
C9—H9B	0.9700	C19—H19B	0.9700
C10—H10A	0.9600	C20—H20A	0.9600
C10—H10B	0.9600	C20—H20B	0.9600
C10—H10C	0.9600	C20—H20C	0.9600
N3—H3A	0.8604	N6—H6A	0.8550
N3—H3B	0.8634	N6—H6B	0.8530
			
N2—C1—N1	112.3 (3)	N5—C11—N4	111.7 (3)
N2—C1—C2	129.3 (3)	N5—C11—C12	128.5 (3)
N1—C1—C2	118.4 (3)	N4—C11—C12	119.8 (3)
C3—C2—N3	124.6 (3)	N6—C12—C13	125.3 (3)
C3—C2—C1	118.0 (3)	N6—C12—C11	117.8 (3)
N3—C2—C1	117.3 (3)	C13—C12—C11	116.9 (3)
C2—C3—C4	120.3 (3)	C12—C13—C14	119.8 (3)
C2—C3—H3	119.8	C12—C13—H13	120.1
C4—C3—H3	119.8	C14—C13—H13	120.1
C5—C4—C3	123.1 (3)	C15—C14—C13	124.2 (3)
C5—C4—Br1	117.5 (3)	C15—C14—Br2	117.1 (3)
C3—C4—Br1	119.5 (3)	C13—C14—Br2	118.6 (3)
C4—C5—N1	116.5 (3)	C14—C15—N4	115.9 (3)
C4—C5—H5	121.7	C14—C15—H15	122.0
N1—C5—H5	121.7	N4—C15—H15	122.0
N1—C6—C7	105.5 (3)	C17—C16—N4	105.1 (3)
N1—C6—H6	127.3	C17—C16—H16	127.4
C7—C6—H6	127.3	N4—C16—H16	127.4
C6—C7—N2	112.0 (3)	C16—C17—N5	112.0 (3)
C6—C7—C8	122.9 (3)	C16—C17—C18	128.3 (3)
N2—C7—C8	125.1 (3)	N5—C17—C18	119.7 (3)
O2—C8—O1	124.4 (3)	O4—C18—O3	124.0 (3)
O2—C8—C7	121.8 (3)	O4—C18—C17	122.9 (3)
O1—C8—C7	113.8 (3)	O3—C18—C17	113.0 (3)
O1—C9—C10	109.4 (3)	C20—C19—O3	111.7 (4)
O1—C9—H9A	109.8	C20—C19—H19A	109.3
C10—C9—H9A	109.8	O3—C19—H19A	109.3
O1—C9—H9B	109.8	C20—C19—H19B	109.3
C10—C9—H9B	109.8	O3—C19—H19B	109.3
H9A—C9—H9B	108.2	H19A—C19—H19B	107.9
C9—C10—H10A	109.5	C19—C20—H20A	109.5
C9—C10—H10B	109.5	C19—C20—H20B	109.5
H10A—C10—H10B	109.5	H20A—C20—H20B	109.5
C9—C10—H10C	109.5	C19—C20—H20C	109.5
H10A—C10—H10C	109.5	H20A—C20—H20C	109.5
H10B—C10—H10C	109.5	H20B—C20—H20C	109.5
C6—N1—C1	106.6 (3)	C11—N4—C16	107.2 (3)
C6—N1—C5	129.8 (3)	C11—N4—C15	123.2 (3)
C1—N1—C5	123.7 (3)	C16—N4—C15	129.6 (3)
C1—N2—C7	103.7 (3)	C11—N5—C17	104.0 (3)
C2—N3—H3A	117.6	C12—N6—H6A	119.8
C2—N3—H3B	118.9	C12—N6—H6B	115.4
H3A—N3—H3B	113.5	H6A—N6—H6B	121.3
C8—O1—C9	114.8 (3)	C18—O3—C19	118.1 (3)

### Hydrogen-bond geometry (Å, °)

**Table:**

*D*—H···*A*	*D*—H	H···*A*	*D*···*A*	*D*—H···*A*
N3—H3A···N5^i^	0.86	2.47	3.300 (4)	161
N3—H3B···O3^ii^	0.86	2.38	3.055 (5)	135
N6—H6B···O2	0.85	2.34	3.096 (4)	147
N6—H6A···O1^iii^	0.86	2.58	3.183 (4)	129
N6—H6A···N2^iii^	0.86	2.60	3.388 (4)	154
C5—H5···O4	0.93	2.26	3.074 (4)	146
C19—H19B···O4	0.97	2.28	2.703 (6)	105

Symmetry codes: (i) −*x*+1, *y*−1/2, −*z*+1/2; (ii) *x*, −*y*+1/2, *z*−1/2; (iii) −*x*+2, *y*+1/2, −*z*+1/2.
